# Toward Precision Imaging: Commentary on the Predictive Value of T2WI and ADC in Prostate Cancer

**DOI:** 10.1590/S1677-5538.IBJU.2025.0305

**Published:** 2025-06-20

**Authors:** Yujie Xu, Hua Xu

**Affiliations:** 1 Yueyang Hospital of Integrated Traditional Chinese and Western Medicine affiliated to Shanghai University of Traditional Chinese Medicine Department of Anesthesiology Shanghai China Department of Anesthesiology, Yueyang Hospital of Integrated Traditional Chinese and Western Medicine affiliated to Shanghai University of Traditional Chinese Medicine, Shanghai, China

To the editor,

We read with great interest the recently published article entitled "[The] (Predictive Value of Multiparametric Magnetic Resonance Imaging (T2-weighted Imaging and Apparent Diffusion Coefficient) for Pathological Grading of Prostate Cancer: a Meta-Analysis)" in the International Brazilian Journal of Urology (1). This well-conducted meta-analysis highlights the increasing value of T2-weighted imaging (T2WI) and apparent diffusion coefficient (ADC) mapping in improving both diagnostic accuracy and cancer detection rates for prostate cancer, and it provides quantitative evidence supporting their integration into clinical practice. The authors are to be commended for synthesizing current evidence and drawing attention to the need for imaging-based grading in risk-adapted prostate cancer management.

However, we wish to offer a few additional observations on limitations not fully addressed in the article, along with suggestions for future research directions. First, the analysis did not stratify diagnostic performance based on tumor location within the prostate. It is well known that the detection sensitivity of T2WI and ADC varies significantly between the peripheral and transitional zones. Tumors in the transitional zone are often confounded by benign prostatic hyperplasia, which can obscure imaging interpretation. Incorporating zone-specific analyses in future studies would allow clinicians to better understand the region-specific strengths and weaknesses of mpMRI and refine biopsy targeting strategies accordingly (2, 3).

Second, the meta-analysis did not adequately address variability in MRI acquisition protocols, hardware, and ADC quantification methods. Differences in scanner vendors, field strengths (1.5T vs. 3.0T), b-values in diffusion imaging, and post-processing techniques can all influence ADC measurements, leading to inter-study inconsistency. Future investigations should advocate for standardized acquisition protocols or include scanner-specific calibration to ensure consistency in quantitative imaging.

Third, while the authors acknowledge the potential of mpMRI, we believe [that] (greater emphasis) should be placed on the integration of artificial intelligence (AI) into diagnostic workflows. AI, particularly deep learning and radiomics approaches, can extract high-dimensional imaging features beyond human perception and combine them with clinical data (e.g., PSA, age, prostate volume) to build robust predictive models. More importantly, AI can enhance reproducibility by reducing inter-observer variability in lesion segmentation and PI-RADS scoring (4, 5). Future studies should explore hybrid models that integrate T2WI, ADC, and clinical variables using explainable AI algorithms trained on large, diverse datasets. This would not only promote individualized risk stratification but may also enable real-time decision support in biopsy-naïve patients.

In conclusion, this meta-analysis provides valuable evidence supporting the diagnostic relevance of T2WI and ADC in prostate cancer grading. To further enhance the clinical utility of mpMRI, we suggest stratifying performance by tumor location, ensuring technical standardization of imaging parameters, and incorporating AI-driven models for improved precision and reproducibility. Such improvements will pave the way for more personalized, efficient, and accurate prostate cancer diagnostics.

Future research should include bigger and more diverse groups with thorough stratification for confounding factors (e.g., gender, baseline anxiety level, medical history) to improve the validity of the results. Furthermore, a longitudinal study assessing the long-term effects of self-visualization on anxiety, pain tolerance, and patient satisfaction over numerous cystoscopy sessions could give additional evidence of its efficacy. Alternative approaches that combine self-visualization with other pain-relieving techniques may also provide useful insights for enhancing the patient experience during cystoscopy. Finally, blinded study design may reduce bias in outcome evaluation, particularly for subjective variables like pain and satisfaction.
